# A Computationally Efficient Approach to Simulate Heart Rate Effects Using a Whole Human Heart Model

**DOI:** 10.3390/bioengineering9080334

**Published:** 2022-07-24

**Authors:** Jiang Yao, Shawn Chen, Julius M. Guccione

**Affiliations:** 1 Dassault Systèmes, Johnston, RI 02919, USA; jiang.yao@3ds.com; 2 Abbott, Sylmar, CA 91342, USA; shawn.chen4@abbott.com; 3 Department of Surgery, University of California at San Francisco, San Francisco, CA 94143, USA

**Keywords:** AV delay, computational modeling, exercise, leadless pacemakers, normal cardiac physiology, regional electromechanics

## Abstract

Computational modeling of the whole human heart has become a valuable tool to evaluate medical devices such as leadless pacemakers, annuloplasty rings and left ventricular assist devices, since it is often difficult to replicate the complex dynamic interactions between the device and human heart in bench-top and animal tests. The Dassault Systèmes Living Heart Human Model (LHHM) is a finite-element model of whole-human-heart electromechanics that has input parameters that were previously calibrated to generate physiological responses in a healthy heart beating at 60 beat/min (resting state). This study demonstrates that, by adjusting only six physiologically meaningful parameters, the LHHM can be recalibrated to generate physiological responses in a healthy heart beating at heart rates ranging from 90–160 beat/min. These parameters are as follows: the sinoatrial node firing period decreases from 0.67 s at 90 bpm to 0.38 s at 160 bpm, atrioventricular delay decreases from 0.122 s at 90 bpm to 0.057 s at 160 bpm, preload increases 3-fold from 90 bpm to 160 bpm, body resistance at 160 bpm is 80% of that at 90 bpm, arterial stiffness at 160 bpm is 3.9 times that at 90 bpm, and a parameter relating myofiber twitch force duration and sarcomere length decreases from 238 ms/mm at 90 bpm to 175 ms/mm at 160 bpm. In addition, this study demonstrates the feasibility of using the LHHM to conduct clinical investigations in AV delay optimization and hemodynamic differences between pacing and exercise. AV delays in the ranges of 40 ms to 250 ms were simulated and stroke volume and systolic blood pressure showed clear peaks at 120 ms for 90 bpm. For a heart during exercise, the increase in cardiac output continues to 160 bpm. However, for a heart during pacing, those physiological parameter adjustments are removed that are related to changes in body oxygen requirements (preload, arterial stiffness and body resistance). Consequently, cardiac output increases initially with heart rate; as the heart rate goes up (>100 bpm), the increasing rate of cardiac output slows down and approaches a plateau.

## 1. Introduction

**Figure 1:**
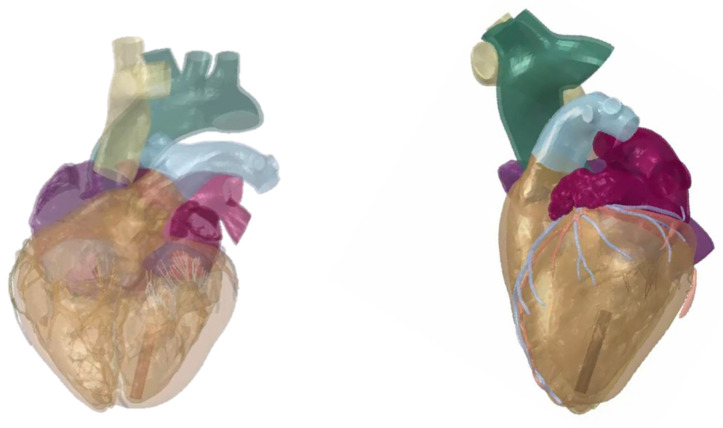
Leadless pacemaker is positioned at the apex region of the left ventricle in LHHM. (**Left**) frontal view, (**Right**) left view.

Continued advances in computational power and methods have enabled whole human heart modeling [1,2,3,4,5] to become a valuable tool for evaluating medical devices, such as leadless pacemakers, annuloplasty rings and left ventricular assist devices, because of the difficulty in replicating the complex dynamic interactions between the device and human heart in bench-top and animal tests. Clinical studies using heart models or that are supplemented with simulations, in comparison to traditional human clinical studies with in vivo measurements, have the potential to save cost and time while reducing risks to patients. The Dassault Systèmes Living Heart Human Model (LHHM) is a finite-element model of the electromechanics of the whole human heart (Figure 1), for which the input parameters were previously calibrated to generate physiological responses in a healthy heart beating in its resting state of 60 beat/min. The dynamic behavior of the LHHM is regulated by realistic mechanical, electrical and fluid physics [1]. It provides a rigorous virtual environment to evaluate the interactions between a medical device and the human heart.

As the heart beats faster, its dynamic interactions with a device are likely to intensify. Moreover, the changes in device–heart interaction [1,2,3,5] may be nonlinear. It is, therefore, critical to calibrate the LHHM for different heart rates to model device–heart interactions where the intensity of motion could be important.

Patients with long term pacemaker implants often develop “pacemaker syndrome”, where patients feel symptomatically worse after pacemaker placement and present with progressively worsening symptoms of congestive heart failure. A multitude of factors could be contributing to this phenomenon, including improper programming of the pacemaker and differences in pacing and natural heart contractions. A computational model that can be used to study the effects of pacemaker settings on strains in the cardiac tissue and the difference between a natural vs. paced heart is a useful tool to help find solutions for pacemaker syndrome.

An example is AV delay. AV delay is the delay in conduction between the atria and ventricles at the AV node [6]. AV delay is optimal when the atria have fully emptied before the ventricles contract [7,8,9]. AV delay must be programmed appropriately for each individual patient with dual-chamber pacing [9]. AV delay has been shown to have a significant effect on hemodynamic performance, and improper adjustment of AV delay can reduce cardiac output by lowering the efficacy of the pacemaker, and it can cause the heart to work harder against elevated back pressure, resulting in increased myocardium strains and development of “pacemaker syndrome” [10,11]. AV delay optimization has been well studied; however, the dependence of optimized AV delay on individual patients is still not well understood.

Additionally, there are other important reasons to consider heart rate in predictive multiscale computational modeling. For example, heart failure with preserved ejection fraction (HFpEF) is considered the greatest unmet need in cardiovascular medicine today because of a general lack of effective treatments. In September 2017, the National Heart, Lung, and Blood Institute convened a two-day working group meeting of experts in HFpEF and novel research methodologies (including machine learning and predictive multiscale computational modeling) to discuss research priorities for HFpEF. Their white paper pointed out the need for improved animal models, including large animal models, which incorporate the effects of exercise [12]. Certainly, it would be impossible to realistically simulate the effects of exercise on HFpEF without a predictive multiscale computational model that can operate at elevated heart rates.

The objectives of this study are to (1) calibrate the LHHM for different heart rates during exercise, (2) study the hemodynamic differences between a natural heart beat during exercise and an artificially paced heart at an elevated heart rate and (3) to evaluate the feasibility of using LHHM in AV delay optimization.

## 2. Methods

Baillargeon et al. [1] created a finite-element model of the whole human heart on the basis of the existing solid model illustrated in Figure 1. This made it possible to model all four chambers as electrically excitable, deformable, hyperelastic, electroactive bodies connected via inflow and outflow conditions of a viscous resistance type [2]. We refer the interested reader to Baillargeon et al. [1] and Sack et al. [2] for the full description of the model, including model features, parameters and the system equations underlying the model. Importantly, significant improvements in the model are described in Baillargeon et al. [13] and Sack et al. [2]. Here, we only describe the changes in the LHHM that were pertinent to this study.

During exercise, sympathetic nerves of the autonomic nervous system increase heart rate, electrical propagation, myocardial contractility and blood flow [14,15]. Exercise also constricts arteries and reduces body resistance [16,17]. Rather than performing a formal optimization of LHHM input parameters to best fit physiological responses in a healthy heart beating at a wide range of heart rates, we first reviewed the literature on physiological responses with an increased heart rate due to exercise and then determined the implications for the LHHM.

We simulated six key effects of exercise from the literature. The corresponding changes we made to the LHHM mimicked neural/hormonal modulations and increased oxygen demand (Table 1 and Table 2). First, autonomic fibers increase the sinoatrial (SA) node firing rate [14], which was simulated by reducing the SA firing period in the LHHM. Second, atrioventricular (AV) node conduction is accelerated [18], which was simulated by reducing the AV delay in the LHHM according to the pre-ejection period measurements using the electrocardiogram for untrained healthy subjects, as reported by Gledhill et al. [19]. Third, heart blood volume increases as the vascular system redistributes blood to tissues with the greatest oxygen demand [20], which was simulated by increasing the preload to increase blood volume into the LHHM. Fourth, systemic vascular resistance falls due to vasodilation of blood vessels in active skeletal muscle [17], which was simulated by reducing body resistance in the LHHM. Fifth, arterial distensibility is decreased due to endothelial and neurohumoral influences on vasodilation [16], which was simulated by increasing arterial stiffness in the LHHM. Lastly, it is widely accepted that myocardial contractility is increased through the Bowditch effect. Several LHHM input parameters can affect myocardial contractility. We chose to simply reduce the value of the parameter that controls myofiber twitch force duration as a function of sarcomere length. In summary, the electrical conduction-related parameters, including SA pacing and AV delay, were prescribed based on the target heart rate and experimental measurements [19]. The selection of hemodynamic parameters (i.e., preload, body resistance, arterial stiffness) and a myocardium contractile parameter, and whether to increase or decrease them, were determined based on the abovementioned physiological changes due to exercise. The absolute magnitudes of the parameters were tuned so that model predictions match the experimental measurements on cardiac output and blood pressure for a cohort of healthy subjects with heart rates of 90 to 160 bpm during excises [19].

After the baseline hemodynamic performance was first established to simulate hearts during exercise, we then studied the hemodynamic differences of hearts during exercise and pacing. For a heart during pacing, heart rate changes are often not associated with excise and changes in body oxygen requirements, and the hemodynamic behaviors would be different for a paced heart from that of a heart during exercise. For a paced heart without oxygen demand increases, the heart model was then simulated with pacing only and those physiological parameters that related to exercise were removed (preload, arterial stiffness and body resistance, Table 3).

To perform AV delay optimization, LHHM were first verified for realistic hemodynamic performance with a standard AV delay (122 ms) at 90 bpm [21], after which a few reduced and prolonged AV delays were introduced into the LHHM.

Hemodynamic outcomes such as blood pressure, pressure of left ventricle and left atrium, stroke volume, cardiac output, etc. were extracted and the trend curves were plotted.

## 3. Results

### 3.1. Results Related to Exercise-Induced Increased Heart Rate

**Figure 2:**
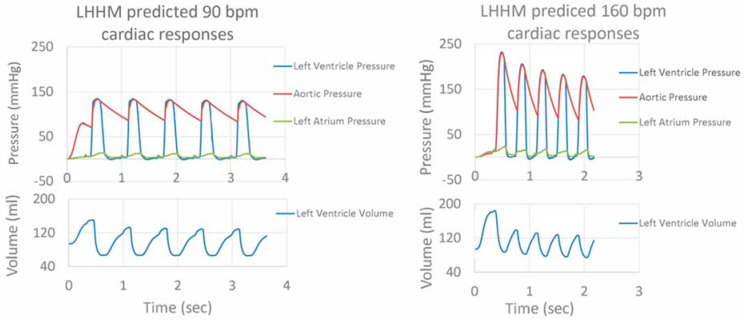
Pressure traces for left ventricle, left atrium and artery, as well as left ventricular volume for five cardiac cycles for simulations of heart rate related to exercise at 90 and 160 beats per minute.

**Figure 3:**
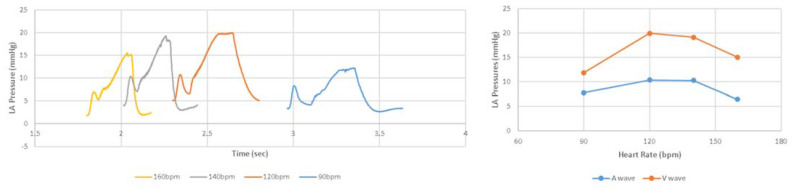
(**Left**) Pressure time traces for left atrium at the fifth cardiac cycle, as well as (**Right**) magnitudes of A wave and V wave left atrium pressures for simulations of heart rate related to exercise at 90 and 160 beats per minute.

After adjustments to the LHHM model inputs (Table 2), cardiac responses equilibrated after five simulated cardiac cycles for all heart rates. The LHHM model behavior when heart rate increased followed the trend reported in Gledhill et al. [19] (Figure 2). Systolic blood pressure increased, diastolic pressure remained unchanged, cardiac output increased, and ventricular ejection time and diastolic filling time were reduced. Absolute differences for cardiac output and blood pressures were within two standard deviations of the mean values of healthy untrained subjects (Figure 2 and Table 4). Left atrium pressure traces at the fifth cardiac cycle for a heart during exercise are plotted for heart rates (90, 120, 140 and 160 bpm) and shown in Figure 3. The A wave and V wave pressures increase from 90 bpm to 120 bpm, plateau up to 140 bpm, and then decrease afterwards.

### 3.2. Results Related to Pacing-Induced Increased Heart Rate

**Figure 4:**
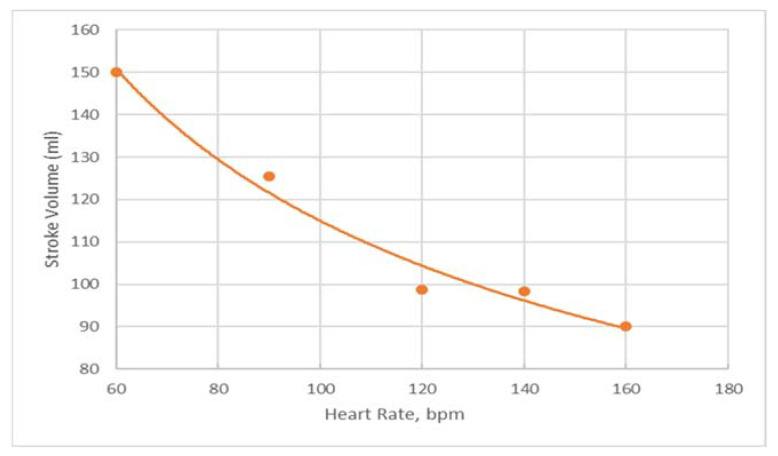
Predictions of stroke volume for paced heart for heart rates of 60, 90, 120, 140 and 160 bpm in orange dots with a trendline fitting the data in orange line.

**Figure 5:**
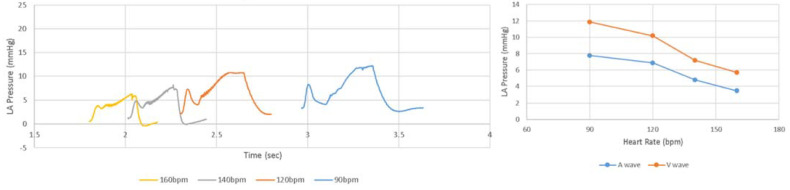
(**Left**) Pressure time traces for left atrium at the fifth cardiac cycle, as well as (**Right**) magnitudes of A wave and V wave left atrium pressures for simulations of heart rate related to pacing at 90 and 160 beats per minute.

When LHHM is configured to simulate pacing without exercise, the model outputs show that the stroke volume reduces with an increasing heart rate (Figure 4). This result is in agreement with in vivo measurements reported in the literature [22]. Left atrium pressure traces at the fifth cardiac cycle for paced hearts are plotted for heart rates (90, 120, 140 and 160 bpm) and shown in Figure 5. The A wave and V wave pressures keep decreasing from 90 bpm to 160 bpm.

**Figure 6:**
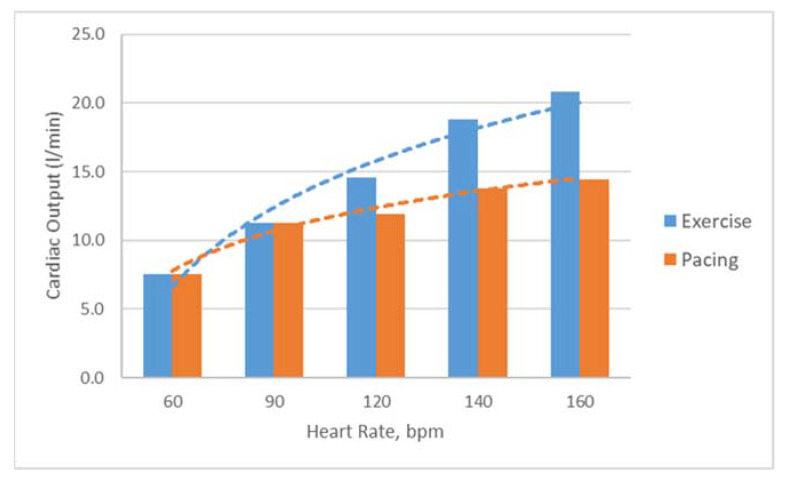
Predictions of cardiac outputs for hearts during exercise and pacing for heart rates of 60, 90, 120, 140 and 160 bpm.

In the LHHM, for a paced heart, cardiac output increases initially with heart rate; as the heart rate goes up (>100 bpm) the rate of CO increase slows down and approaches a plateau; for a heart during exercise, the CO increase continues to become a higher heart rate (Figure 6). These results are in agreement, as reported in the literature [23,24]. Other parameters, such as LV ejection time and filling time, also follow the trend as measured in vivo [21].

### 3.3. Results Related to Optimized AV Delay

**Figure 7:**
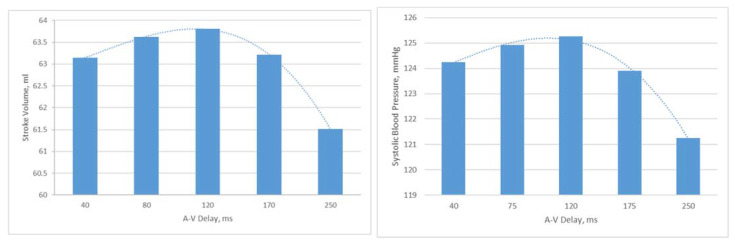
(**Left**) Stroke volume vs. AV delay predicted by LHHM with heart rate of 90 bpm, (**Right**) systolic blood pressure vs. AV delay predicted by LHHM with heart rate of 90 bpm.

**Figure 8:**
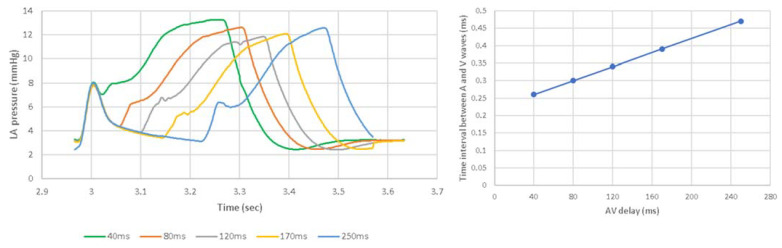
(**Left**) Pressure time traces for left atrium at the fifth cardiac cycle. (**Right**) Time interval between the peaks of LA pressures at A wave and V wave with heart rate of 90 bpm and AV delay of 40, 80, 120, 170, 250 ms.

AV delays in the range of 40 ms to 250 ms were simulated in the Living Heart Human Model. The results on stroke volume show a clear peak at 120 ms in the LHHM at 90 bpm (Figure 7), which agrees with the values reported in the literature [22]. Systolic blood pressure also shows a clear peak at 120 ms AV delay [25] (Figure 7), which agrees with the study conducted by Manisty. The absolute changes in stroke volume reported by the LHHM is less than that reported in the literature [22]. Left atrium pressure traces at the fifth cardiac cycle for all AV delay simulations are shown in Figure 8. The time interval between the peaks of A wave and V wave pressures increases linearly as the AV delay increases.

## 4. Discussion

Our results demonstrate the feasibility of using the LHHM to simulate the consequences of an elevated heart rate, i.e., increase in cardiac output, increase in blood pressure and decrease in cardiac time intervals. We also demonstrated that the model can differentiate between a pacing- and exercise-induced elevated heart rate. Due to the lack of neural/hormonal modulations and lack of increased oxygen demand during pacing, when the heart rate goes up, the cardiac output increase slows down and approaches a plateau, whereas during exercise, the cardiac output continues to increase to a higher heart rate. This study also demonstrates the feasibility of using the LHHM to optimize AV delay to achieve maximal stroke volume and systolic blood pressure.

This model provides a virtual environment to evaluate device–heart interaction at an elevated heart rate. Already, we have used the LHHM to simulate the effects of very different annuloplasty rings for correcting mitral regurgitation [3,4,5] and of a left ventricular assist device for treating acute heart failure [2]. Now, we have a very straightforward approach (i.e., adjusting only six physiologically meaningful parameters) to simulate the effect of an elevated heart rate on the efficacy of these and other devices.

Our study is among the very few published that couple the left and right heart, including atria and ventricles, to the rest of the systemic and pulmonary circulations [26,27]. Our heart geometry is from a segmentation of a healthy subject and is, therefore, more realistic than those models that oversimplify the anatomy; for example, [28,29]. A few other investigators have used similar whole heart geometry [26,27], but ours is the first investigation to simulate regional mechanics in a range of heart rates and compare results to experimental or clinical data.

### 4.1. Comparison to Other Models for Simulating Heart Rate Effects

To the best of our knowledge, ours is the first study to use a whole human heart model to simulate the effects of heart rate on ventricular mechanics. A recent example of a finite-element model used to simulate heart rate effects on cardiac mechanics is the study by Sturla et al. [30] on the effects of the Mitraclip system on mitral valve function. To simulate heart rate effects on systolic excursion of the mitral valve leaflets into the left atrial chamber at the peak of systolic trans-mitral pressure (40, 60, 80 and 100 beats per minute), they simply specified the left atrial and left ventricular pressures; they did not use a cardiac model, or even a lumped-parameter circulatory model to compute those pressures. Using computational fluid dynamics simulations, Jahandardoost et al. [31] studied the effect of heart rate on the hemodynamics of a bileaflet mechanical heart valve that was implanted in the aortic position. They studied 60–150 bpm with idealistic boundary conditions of a pressure-based inlet and an outlet mass flow rate; their studies also lacked realistic boundary conditions from a full heart model.

### 4.2. Parameter Estimation and Machine Learning

It would not have been practical to optimize every parameter in the LHHM in order to best fit (for example, in a least-squares sense) measurements of hemodynamics and cardiac output at different elevated heart rates. The total number of variables in the LHHM is 196, with 57 parameters associated with the electrical model, 132 parameters associated with the excitation-contraction coupling model and 7 parameters associated with the systemic blood circulation model. In a recent machine learning study [32], we estimated that it would take thousands of CPU hours to tune the LHHM. In that study, only six parameters defining ventricular mechanical properties of the LHHM were altered using an optimal Latin hypercube design of the experiments to obtain “training” of the finite-element models with varied ventricular pressures and volumes (all corresponding to a heart rate of 60 beats per minute). The number of selected finite-element models for training and test data were 77 and 3, respectively. The finite-element models were used to create machine learning models that reduced left ventricular pressure and volume prediction time from nearly 1000 CPU hours to 11 CPU seconds. A similar study could be performed to also greatly reduce hemodynamic and cardiac output prediction time for a range of elevated heart rates.

### 4.3. Limitations

Perhaps the greatest limitation of our modeling study of the whole human heart is that we used a time-varying elastance approach, rather than a cellular model approach, to simulate electromechanical coupling. There are great savings in computation time when a time-varying elastance approach, in which active stress is an analytic function of time since the onset of contraction, intracellular calcium concentration and sarcomere length, is used instead of a cellular model approach in which, for example, active stress is computed from a system of 30 or more ordinary differential equations [33], especially since it typically takes five cardiac cycles for our whole heart model simulation to reach a steady state. We demonstrated previously that time-varying models of left ventricular and biventricular regional mechanics are realistic [2,34]. Another potential limitation is our whole heart model is able to realistically simulate clinical observations of the effects of heart rate on stroke volume, but not necessarily also on end-diastolic and end-systolic volumes, which are more difficult to measure clinically.

## 5. Conclusions

This study demonstrates that by calibrating only six physiologically meaningful parameters, a computational model of a whole human model can accurately capture physiological responses of a healthy heart at heart rates ranging from 90–160 bpm. The results demonstrate the feasibility of using the heart model (LHHM) to conduct clinical investigations in AV delay optimizations and in studying the hemodynamic differences between hearts during exercise and pacing. The outputs from the LHHM, in general, are in good agreement with in vivo measurements, although some parameters show the correct trends but with some quantitative differences. Our model is useful to evaluate medical devices that allow complex dynamic interactions between the device and human heart to be considered, which is difficult to replicate in bench-top and animal tests.

Future development plans include continuing to improve the fidelity of the LHHM to include more complex heart physiologies that are critical for providing a realistic boundary condition for device testing and evaluation. The current living heart model includes some complex and nonlinear physiologies, such as active tissue material modeling intended to capture the Frank–Starling effect (i.e., the strength of the heart’s systolic contract is directly proportional to its diastolic expansion) [2]. Advanced features in the LHHM enable further enhancements. For example, the atria and ventricle components of the model make it possible to study atrioventricular coupling [35], but further tuning and calibration are required to model this atrioventricular coupling more accurately. Another example is the fiber-reinforced formulation for a passive material model that enables modeling of ventricular compliance [1]. Future work can focus on calibrating the compliance to capture diseased conditions, such as increased compliance for dilated cardiomyopathy or decreased compliance for ventricular hypertrophy [15]. Currently, the model only considers forward systolic flow from left ventricle to artery. It does not include the reflected wave from the peripheral arterial branch back to the heart. Including this feature could help enhance model fidelity in arterial physiology and pathophysiology [36]. For heart failure with preserved ejection fraction, a possible future study could relate clinical presentations (i.e., LV hypertrophy, impaired LV systolic performance, LA remodeling, etc.) and underlying pathophysiologies of HFpEF (i.e., prolonged isovolumetric LV relaxation, slow LV filling, increased LV diastolic stiffness, transmural changes in LV myocardial contraction, increased arterial and venous resistance, etc.) using computational simulations [37,38,39]. With these improvements, the LHHM has the potential to become a valuable platform to conduct more sophisticated clinical studies with simulations instead of in vivo human trials.

## Figures and Tables

**Table 1:** Adjustments to LHHM model inputs to study increased heart rate during exercise.

Physiological Responses with Increased Heart Rate Due to Exercise	Implication for LHHM
Autonomic fibers increase SA firing rate	Reduce SA firing period
AV conduction accelerates	Reduce AV delay according to pre-ejection period on ECG
Myocardium contractility increases through the Bowditch effect	Reduce the “m” parameter that controls sarcomere relaxation duration
Heart blood volume increases as vascular system redistributes blood to tissues with greatest demand for oxygen	Increase preload to increase blood volume into heart
Systemic vascular resistance falls due to vasodilatation of blood vessels in active skeletal muscles	Reduce body resistance
Arterial distensibility decreases due to endothelial and neurohumoral influences in vasodilatation	Increase arterial stiffness

**Table 2:** Adjustment to LHHM model inputs to study increased heart rate during exercise.

Heart rates (bpm)	90	120	140	160
SA firing period (second)	0.67	0.50	0.43	0.38
AV delay (second)	0.122	0.087	0.068	0.057
m (ms/mm)	238	238	175	175
Preload (×Baseline)	1.0	2.0	3.0	3.0
Body resistance (×Baseline)	1.0	0.9	0.7	0.8
Arterial stiffness (×Baseline)	1.0	1.5	2.0	3.9

**Table 3:** Adjustment to LHHM model inputs to study increased heart rate during pacing.

Heart rates (bpm)	90	120	140	160
SA firing period (second)	0.67	0.50	0.43	0.38
AV delay (second)	0.122	0.087	0.068	0.057
m (ms/mm)	238	238	175	175
Preload (×Baseline)	1.0	1.0	1.0	1.0
Body resistance (×Baseline)	1.0	1.0	1.0	1.0
Arterial stiffness (×Baseline)	1.0	1.0	1.0	1.0

**Table 4:** Cardiac output, left ventricular ejection time, diastolic filling time and blood pressure for each heart rate predicted by LHHM and experimentally measured on untrained healthy subjects by Gledhill et al. [19].

Measurement	Source	Heart Rate (bpm)			
		90	120	140	160
Cardiac Output (L min^−1^)	LHHM	11.3	14.6	18.8	20.8
	Gledhill	10.8 ± 0.4	15.1 ± 0.4	17.8 ± 0.6	20.2 ± 0.9
Cardiac Time Interval (ms)					
Left Ventricle Ejection Time	LHHM	228	213	187	163
	Gledhill	212 ± 11	208 ± 4	198 ± 2	175 ± 3
Diastolic Filling Time	LHHM	317	200	174	155
	Gledhill	342 ± 55	204 ± 9	185 ± 4	168 ± 4
Blood Pressure (mmHg)					
Diastolic	LHHM	83	82	81	81
	Gledhill	80 ± 2	79 ± 3	81 ± 2	82 ± 3
Systolic	LHHM	129	141	159	178
	Gledhill	135 ± 3	143±2	158 ± 4	172 ± 3

## Data Availability

The data presented in this study can be regenerated by licensing the publically accessible Living Heart Model, licensed by Dassault Systèmes by making these changes reported in this study.
